# Indomethacin restrains cytoplasmic nucleic acid-stimulated immune responses by inhibiting the nuclear translocation of IRF3

**DOI:** 10.1093/jmcb/mjae015

**Published:** 2024-04-05

**Authors:** Miao Wang, Xiao-Wei Li, Sen-Chao Yuan, Jie Pan, Zeng-Lin Guo, Li-Ming Sun, Shao-Zhen Jiang, Ming Zhao, Wen Xue, Hong Cai, Lin Gu, Dan Luo, Ling Chen, Xue-Qing Zhou, Qiu-Ying Han, Jin Li, Tao Zhou, Tian Xia, Tao Li

**Affiliations:** Nanhu Laboratory, National Center of Biomedical Analysis, Beijing 100850, China; Nanhu Laboratory, National Center of Biomedical Analysis, Beijing 100850, China; Nanhu Laboratory, National Center of Biomedical Analysis, Beijing 100850, China; Nanhu Laboratory, National Center of Biomedical Analysis, Beijing 100850, China; Nanhu Laboratory, National Center of Biomedical Analysis, Beijing 100850, China; Nanhu Laboratory, National Center of Biomedical Analysis, Beijing 100850, China; Nanhu Laboratory, National Center of Biomedical Analysis, Beijing 100850, China; School of Basic Medical Sciences, Fudan University, Shanghai 200032, China; Nanhu Laboratory, National Center of Biomedical Analysis, Beijing 100850, China; Nanhu Laboratory, National Center of Biomedical Analysis, Beijing 100850, China; Nanhu Laboratory, National Center of Biomedical Analysis, Beijing 100850, China; Nanhu Laboratory, National Center of Biomedical Analysis, Beijing 100850, China; Nanhu Laboratory, National Center of Biomedical Analysis, Beijing 100850, China; School of Medicine, Tsinghua University, Beijing 100084, China; Nanhu Laboratory, National Center of Biomedical Analysis, Beijing 100850, China; School of Medicine, Tsinghua University, Beijing 100084, China; Nanhu Laboratory, National Center of Biomedical Analysis, Beijing 100850, China; Nanhu Laboratory, National Center of Biomedical Analysis, Beijing 100850, China; Nanhu Laboratory, National Center of Biomedical Analysis, Beijing 100850, China; Nanhu Laboratory, National Center of Biomedical Analysis, Beijing 100850, China; Nanhu Laboratory, National Center of Biomedical Analysis, Beijing 100850, China; Nanhu Laboratory, National Center of Biomedical Analysis, Beijing 100850, China; School of Basic Medical Sciences, Fudan University, Shanghai 200032, China

**Keywords:** indomethacin, IRF3 nuclear translocation, autoimmune diseases

## Abstract

The recognition of cytosolic nucleic acid triggers the DNA/RNA sensor–IRF3 axis-mediated production of type I interferons (IFNs), which are essential for antiviral immune responses. However, the inappropriate activation of these signaling pathways is implicated in autoimmune conditions. Here, we report that indomethacin, a widely used nonsteroidal anti-inflammatory drug, inhibits nucleic acid-triggered IFN production. We found that both DNA- and RNA-stimulated IFN expression can be effectively blocked by indomethacin. Interestingly, indomethacin also prohibits the nuclear translocation of IRF3 following cytosolic nucleic acid recognition. Importantly, in cell lines and a mouse model of Aicardi–Goutières syndrome, indomethacin administration blunts self-DNA-induced autoimmune responses. Thus, our study reveals a previously unknown function of indomethacin and provides a potential treatment for cytosolic nucleic acid-stimulated autoimmunity.

## Introduction

Type I interferons (IFNs) play a central role in the immune defense against pathogen infections. The recognition of pathogen-derived nucleic acids by pattern-recognition receptors is one of the major events that trigger the production of IFN. Although IFN is important for mediating the anti-infection immune response, its excessive production can result in tissue damage and the development of autoimmunity. Therefore, the concept of type I interferonopathies is established to include a broader set of human disorders, in which the constitutive production of type I IFN is a common characteristic and directly related to pathogenesis (Crow, 2011; Lee-Kirsch, 2017; Crow and Stetson, 2022).

Upon DNA virus infection, the recognition of cytosolic DNA is a critical mechanism for host defense. Cyclic guanosine monophosphate–adenosine monophosphate (cGAMP) synthase (cGAS) has been demonstrated to be a key sensor for the recognition of cytoplasmic DNA. Upon the appearance of DNA in the cytoplasm, cGAS is activated and produces cGAMP to activate stimulator of interferon genes (STING) and the downstream signaling. This activation results in the expression of proinflammatory cytokines, including IFN-β and interleukins, triggering robust immune responses (Gao et al., 2013; Sun et al., 2013; Wu et al., 2013). Similar to cGAS, retinoic acid-inducible gene-I (RIG-I)-like receptors (RLRs) are known to play a crucial role in cytoplasmic RNA recognition. Two members of the RLR family, RIG-I and melanoma differentiation-associated protein 5 (MDA5), serve as sensors for abnormal cytosolic RNAs (Yoneyama et al., 2004; Kawai et al., 2005; Hornung et al., 2006; Kato et al., 2006; Goubau et al., 2014). The engagement of DNA/RNA sensors potentiates the phosphorylation and activation of the downstream transcription factor interferon regulatory factor 3 (IRF3). This activation leads to the formation of IRF3 homodimers and subsequent nuclear translocation (Lin et al., 1998, 1999). Once present in the nucleus, IRF3 homodimers bind to IFN-stimulated response element to initiate IFN transcription (Levy et al., 1988; Tanaka et al., 1993; Fujii et al., 1999; Honda et al., 2006; Tamura et al., 2008).

The accumulation of abnormal DNA or RNA in the cytoplasm has been observed in various human disorders (Ablasser et al., 2013), with Aicardi-Goutières syndrome (AGS) as an example. Mutations in several crucial factors involved in nucleic acid-sensing pathways have been identified in AGS patients (Crow et al., 2006a, b). For example, loss-of-function mutations in DNA 3′ repair exonuclease 1 (TREX1) result in the accumulation of cytoplasmic DNA, which chronically stimulates the cGAS–STING pathway (Ablasser et al., 2014; Gao et al., 2015; Gray et al., 2015). In addition, the *ADAR* gene encodes an enzyme known as adenosine deaminase acting on RNA (ADAR1), which can edit RNA by converting adenosine into inosine (Bass, 2002). Mutations in *ADAR* result in the accumulation of unedited endogenous RNAs, which act as danger signals that stimulate MDA5 and induce the production of type I IFN (Liddicoat et al., 2015). Improper activation of these pathways by endogenous nucleic acids triggers autoimmune responses in human disorders.

Nonsteroidal anti-inflammatory drugs (NSAIDs) are widely used to relieve pain and control inflammation in humans (Brooks and Day, 1991; Kean and Buchanan, 2005; Rainsford, 2007). Although the inhibition of cyclooxygenase (COX) is the well-known mechanism of action for NSAIDs (Funk, 2001), several COX-independent effects have been reported. For example, indomethacin has been shown to activate the NRF2 signaling pathway (Eisenstein et al., 2022). The suppression of NF-κB by aspirin and the inhibition of the inflammasome by ibuprofen were also observed (Yin et al., 1998; Smith et al., 2017). We recently reported that aspirin inhibits cGAS activation through acetylation and thus exhibits promising effects in treating AGS (Dai et al., 2019). In the current study, we further screened 24 NSAIDs from different NSAID categories to assess their activities in inhibiting IFN production. Interestingly, we found that indomethacin, a nonselective inhibitor of COX-1 and COX-2, potently suppressed both cytosolic DNA- and RNA-induced IFN expression. Mechanistic studies revealed that indomethacin interfered with the nuclear translocation of IRF3. Notably, we also showed that indomethacin ameliorated autoimmune responses in AGS mice. Our findings indicate the potential of using indomethacin to treat cytosolic nucleic acid-related autoimmune diseases.

## Results

### The inhibition of HT-DNA-stimulated IFN expression by NSAIDs

**Figure 1:**
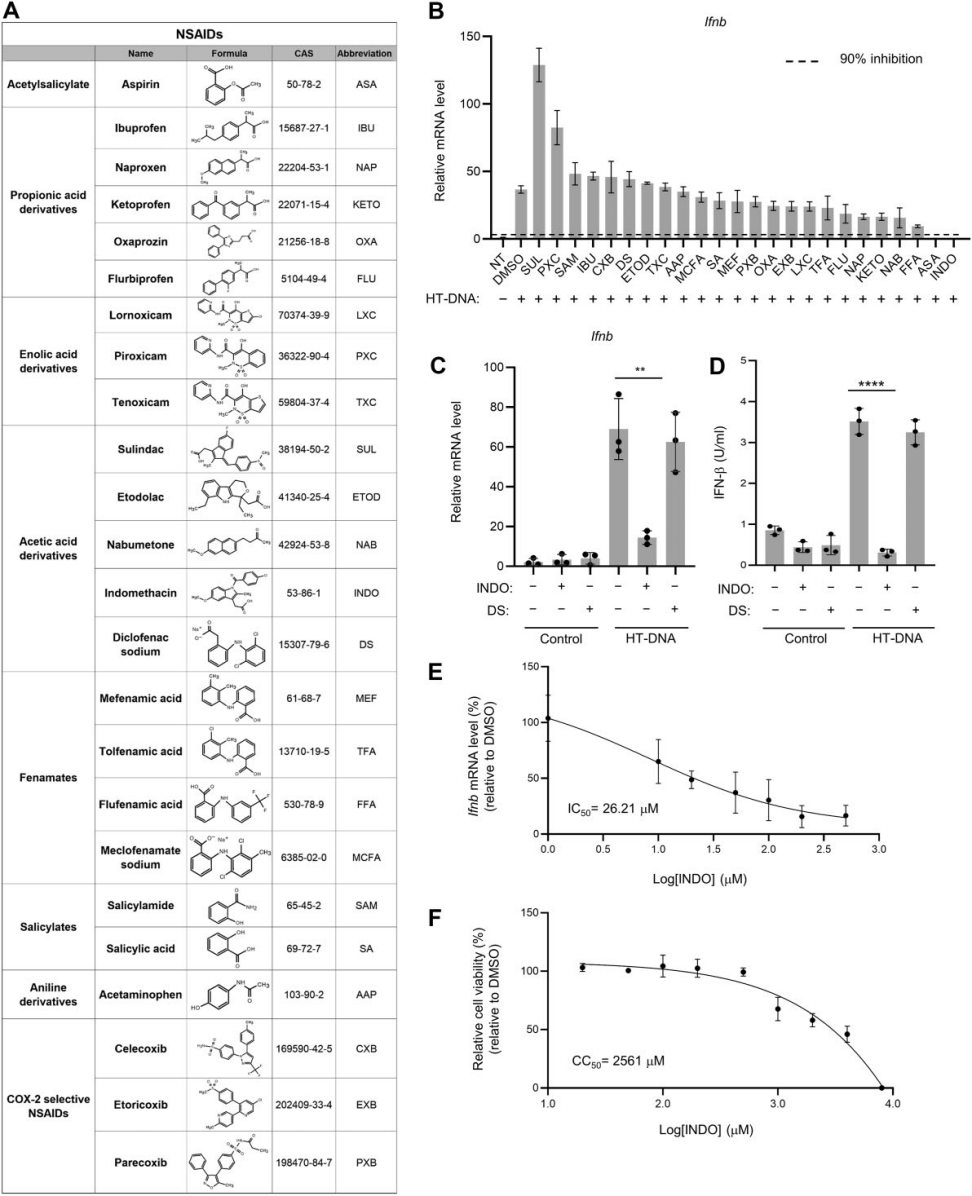
The inhibition of HT-DNA-stimulated IFN expression by NSAIDs. (**A**) Twenty-four selected NSAIDs from eight major categories. (**B**) Relative mRNA levels of *Ifnb* were analyzed by qPCR in L929 cells stimulated with HT-DNA (1 μg/ml) after a 6-h pretreatment with the indicated NSAIDs. (**C** and **D**) L929 cells were treated with diclofenac sodium or indomethacin (200 μM), followed by HT-DNA (1 μg/ml) stimulation. (**C**) Relative *Ifnb* mRNA levels were analyzed by qPCR. (**D**) The secreted IFN-β levels were measured by ELISA. (**E**) L929 cells were treated with indomethacin at the indicated concentrations for 24 h, followed by HT-DNA stimulation. The mRNA levels of *Ifnb* were analyzed by qPCR. The IC_50_ of indomethacin was calculated by Statistical Package for the Social Sciences (SPSS). (**F**) L929 cells were treated with indomethacin at the indicated concentrations for 24 h. Cell viability was analyzed by the MTS assay. The CC_50_ was calculated by SPSS. *n* = 3 technical replicates (**B, E**, and **F**) or *n* = 3 biological replicates (**C** and **D**). Data are presented as mean ± SD. **P < 0.01, ****P < 0.0001, Student's *t*-test.

To examine the potential effect of NSAIDs on cytoplasmic DNA-triggered IFN expression, we prepared a small-scale library that consisted of 24 selected NSAIDs. These drugs were chosen from eight major categories of NSAIDs (Brooks and Day, 1991), including acetylsalicylate, propionic acid derivatives, enolic acid derivatives, acetic acid derivatives, fenamates, salicylates, aniline derivatives, and COX-2 selective NSAIDs (Figure 1A). We also took the suitability for children into account, as some of the nucleic acid-induced autoimmune diseases are childhood-onset diseases, such as AGS and STING-associated vasculopathy with onset in infancy (SAVI) (Jeremiah et al., 2014; Liu et al., 2014). L929 cells (murine fibroblasts) were treated with the selected NSAIDs for 6 h, followed by transfection of herring testis DNA (HT-DNA) as an intracellular DNA mimic, and the *Ifnb* expression levels were determined by quantitative polymerase chain reaction (qPCR). Among the 24 NSAIDs tested, indomethacin most significantly suppressed HT-DNA-induced *Ifnb* expression (Figure 1B), similar to the positive control aspirin (Dai et al., 2019). Another experiment using diclofenac sodium as a negative control showed that indomethacin suppressed HT-DNA-stimulated *Ifnb* expression and IFN-β level, as determined by qPCR and enzyme-linked immunosorbent assay (ELISA), respectively (Figure 1C and D). Both diclofenac sodium and indomethacin belong to the acetic acid derivative category (Brooks and Day, 1991). Our data suggested the relatively specific effect of indomethacin on the inhibition of intracellular nucleic acid sensing and also ruled out the possibility that indomethacin inhibits nucleic acid-induced IFN production through suppressing COX. We further assessed the efficiency and cytotoxicity of indomethacin by performing dose–response experiments and determined its half-maximal inhibitory concentration (IC_50_) and half-maximal cytotoxicity concentration (CC_50_) at 26.21 μM and 2561 μM, respectively (Figure 1E and F).

### Indomethacin inhibits cytosolic DNA- and viral DNA-induced type I IFN expression

**Figure 2:**
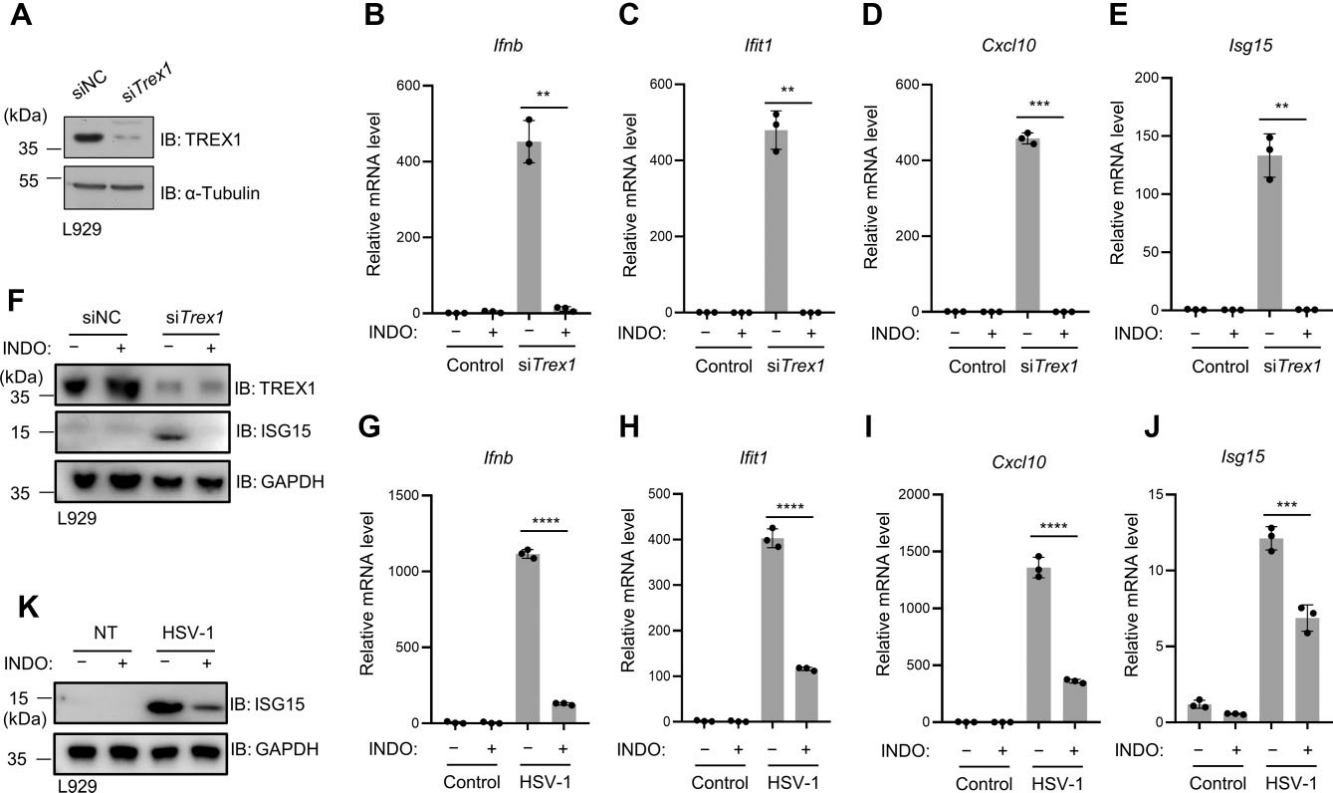
Indomethacin inhibits cytosolic DNA- and viral DNA-induced type I IFN expression. (**A**) Knockdown of TREX1 in L929 cells was examined by western blotting. (**B**–**F**) Control or *Trex1*-knockdown L929 cells were treated with or without indomethacin (200 μM) for 6 h. (**B**–**E**) Relative mRNA levels of *Ifnb* and ISGs were analyzed by qPCR. (**F**) Immunoblotting analysis of ISG15 protein. (**G**–**K**) L929 cells were infected with HSV-1 (MOI = 10) after a 6-h pretreatment with or without indomethacin (200 μM). (**G**–**J**) Relative mRNA levels of *Ifnb* and the indicated ISGs. (**K**) Immunoblotting analysis of ISG15 protein. *n* = 3 biological replicates. Data are presented as mean ± SD. **P < 0.01, ***P < 0.001, ****P < 0.0001, Welch's *t*-test (**B**–**E**) or Student's *t*-test (**G**–**J**).

We next turned to self-DNA-stimulated immune responses. TREX1 is a DNase responsible for degrading cytosolic DNA (Mazur and Perrino, 1999; Morita et al., 2004) and plays a crucial role in preventing self-DNA-induced autoimmunity (Stetson et al., 2008). Loss-of-function mutations of TREX1 lead to the accumulation of self-DNA, which constantly activates the cGAS–STING pathway, and subsequent promotion of autoimmune responses (Ablasser et al., 2014; Gao et al., 2015; Gray et al., 2015). Using siRNAs, we generated *Trex1*-knockdown L929 cells and confirmed TREX1 deficiency by immunoblotting (Figure 2A). We found that the elevated transcription levels of IFN and interferon-stimulated genes (ISGs) in *Trex1*-knockdown cells were significantly inhibited by indomethacin (Figure 2B–E), and the enhanced ISG15 protein level was also suppressed (Figure 2F). Consistently, in L929 cells infected with herpes simplex virus 1 (HSV-1), HSV-1-induced *Ifnb* and ISG expression was also attenuated by indomethacin (Figure 2G–K). Thus, indomethacin is effective in suppressing cytosolic and viral DNA-induced IFN expression.

### Indomethacin inhibits RNA-induced type I IFN expression

**Figure 3:**
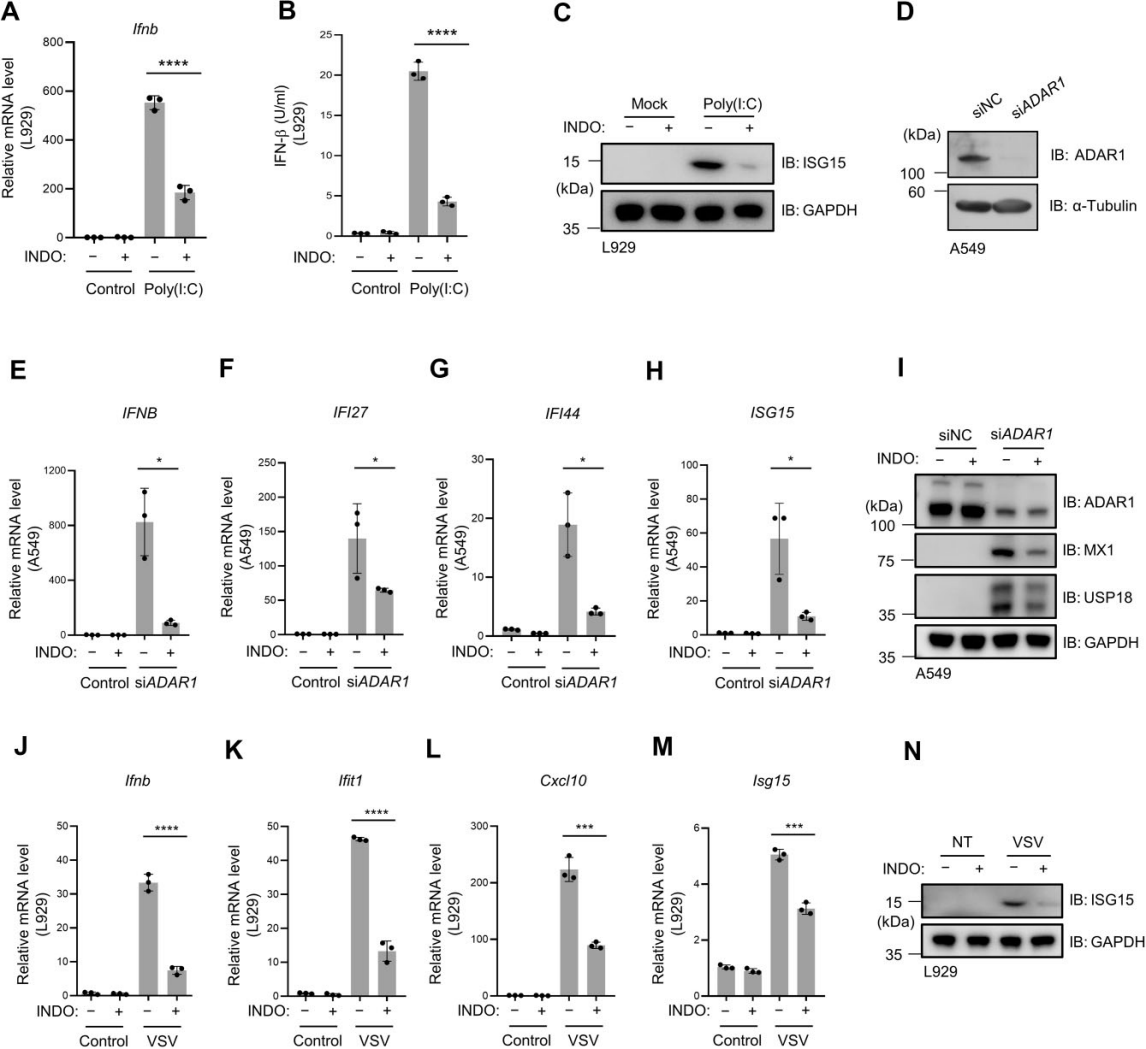
Indomethacin inhibits RNA-induced type I IFN expression. (**A**–**C**) L929 cells were treated with or without indomethacin, followed by poly(I:C) (1 μg/ml) transfection. (**A**) Relative *Ifnb* mRNA levels were analyzed by qPCR. (**B**) The secreted IFN-β levels were measured by ELISA. (**C**) Immunoblotting analysis of ISG15 protein expression. (**D**) Knockdown of ADAR1 in A549 cells was examined by western blotting. (**E**–**I**) Control or *ADAR1*-knockdown A549 cells were treated with or without indomethacin (200 μM) for 6 h. (**E**–**H**) Relative mRNA levels of *IFNB* and indicated ISGs. (**I**) Immunoblotting analysis of protein levels of the indicated ISGs. (**J**–**N**) L929 cells were infected with VSV (MOI = 1) after a 6-h pretreatment with or without indomethacin (200 μM). (**J**–**M**) Relative mRNA levels of *Ifnb* and the indicated ISGs. (**N**) Immunoblotting analysis of the ISG15 protein. *n* = 3 biological replicates. Data are presented as mean ± SD. *P < 0.05, ***P < 0.001, ****P < 0.0001, Welch's *t*-test (**E, G**, and **H**) or Student's *t*-test (**A, B, F**, and **J**–**M**).

To determine whether indomethacin affects the intracellular RNA-induced IFN expression, we used the dsRNA polyinosinic-polycytidylic acid [poly(I:C)] to induce *Ifnb* expression in L929 cells, and the following indomethacin treatment inhibited poly(I:C)-stimulated IFN-β and ISG15 expression (Figure 3A–C). In AGS patients, mutations in key factors in the intracellular RNA-processing pathway have been reported (Crow et al., 2006b; Rice et al., 2012, 2014; Liddicoat et al., 2015). For example, ADAR1 is encoded by the *ADAR* gene and functions as a deaminase, converting adenosine to inosine (Bass, 2002). The adenosine-to-inosine editing process prevents endogenous RNAs from forming long double-stranded helix regions that stimulate the MDA5–MAVS pathway (Liddicoat et al., 2015). In mice, the deletion of *Adar* is embryonically lethal due to the excessive production of type I IFN (Hartner et al., 2009). Using siRNAs, we knocked down the expression of ADAR in A549 cells (Figure 3D) and observed a significant increase in *IFNB* expression (Figure 3E). Indomethacin treatment significantly reduced the expression levels of *IFNB* and ISGs (Figure 3E–I). Additionally, in L929 cells infected with vesicular stomatitis virus (VSV), we observed that VSV-induced *Ifnb* and ISG expression was dampened by indomethacin (Figure 3J–N). Taken together, our data indicate that indomethacin is effective in treating both cytosolic DNA- and RNA-induced immune responses.

### Indomethacin inhibits nucleic acid-induced IFN expression in human immune cells

**Figure 4:**
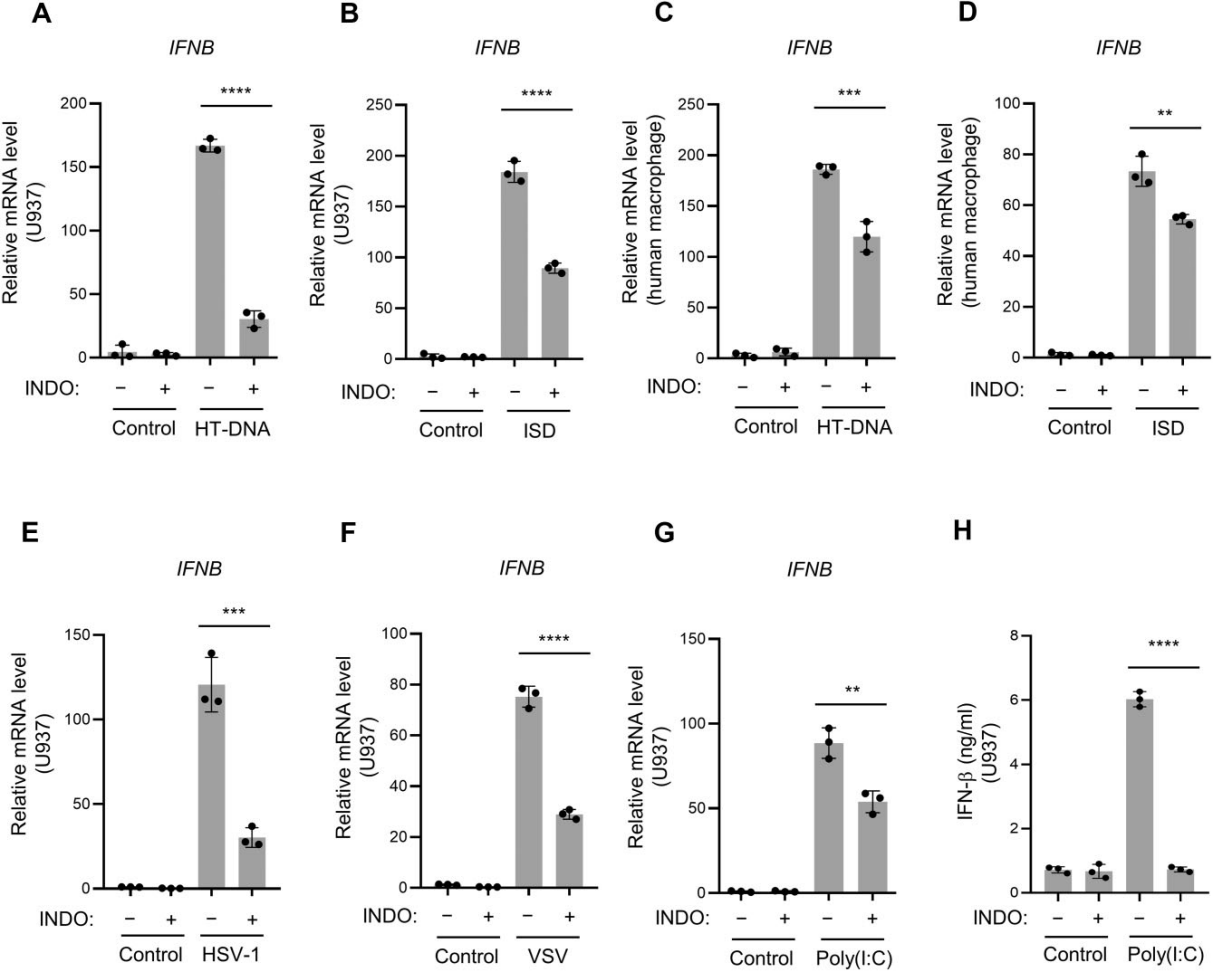
Indomethacin inhibits nucleic acid-induced IFN expression in human immune cells. All the cells were pretreated with or without indomethacin (200 μM) for 6 h before the indicated nucleic acid stimulation. (**A** and **B**) Relative mRNA levels of *IFNB* were analyzed by qPCR in U937 cells stimulated with HT-DNA or ISD. (**C** and **D**) Relative mRNA levels of *IFNB* in PBMC-derived macrophages stimulated with HT-DNA or ISD. (**E** and **F**) Relative mRNA levels of *IFNB* in U937 cells upon HSV-1 or VSV infection. (**G** and **H**) Relative *IFNB* mRNA levels (**G**) and the secreted IFN-β levels measured by ELISA (**H**) in U937 cells transfected with poly(I:C). *n* = 3 biological replicates. Data are presented as mean ± SD. **P < 0.01, ***P < 0.001, ****P < 0.0001, Student's *t*-test.

We further confirm the above findings in human immune cells. Either HT-DNA- or interferon stimulatory DNA (ISD)-elevated *IFNB* mRNA level was suppressed by indomethacin in the monocytic U937 cells (Figure 4A and B) and peripheral blood mononuclear cells (PBMCs)-derived human primary macrophages (Figure 4C and D). Indomethacin also suppressed IFN expression triggered by HSV-1 infection, VSV infection, or poly(I:C) transfection in U937 cells (Figure 4E–H). Thus, indomethacin inhibits both DNA- and RNA-induced type I IFN in human immune cells.

### Indomethacin suppresses nucleic acid-stimulated type I IFN through preventing the nuclear translocation of IRF3

**Figure 5:**
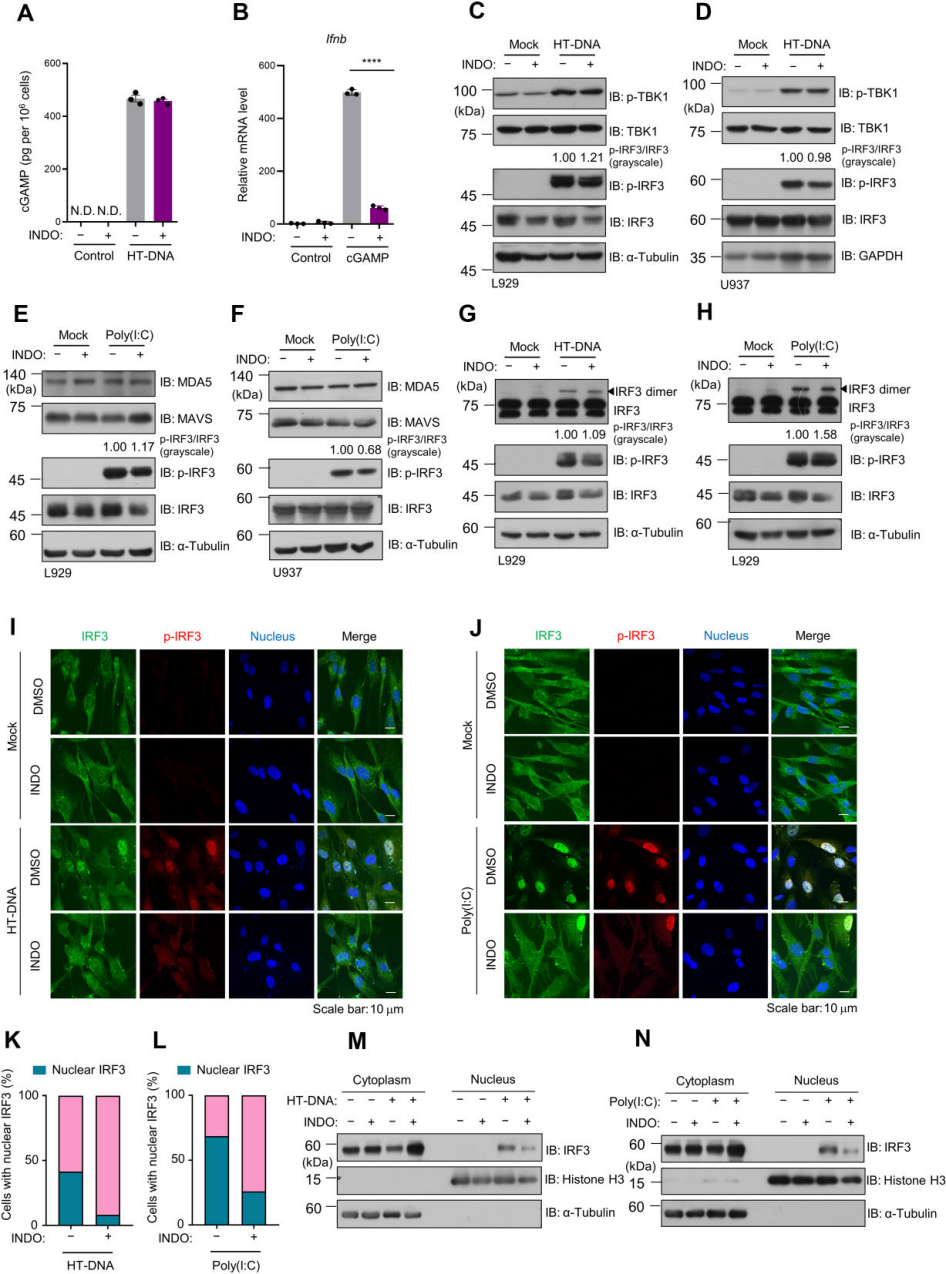
Indomethacin suppresses nucleic acid-stimulated type I IFN through preventing the nuclear translocation of IRF3. All the cells were pretreated with or without indomethacin (200 μM) for 6 h before the indicated nucleic acid stimulation. (**A**) cGAMP production in L929 cells under HT-DNA (2 μg/ml) stimulation was analyzed by liquid chromatography–mass spectrometry/multiple reaction monitoring. *n* = 3 biological replicates. N.D., not detected. (**B**) Relative *Ifnb* mRNA levels were analyzed by qPCR in L929 cells under cGAMP (1 μg/ml) stimulation. *n* = 3 biological replicates. Data are presented as mean ± SD. ****P < 0.0001, Student's *t*-test. (**C** and **D**) Immuno- blotting analysis of the indicated proteins in L929 and U937 cells stimulated with HT-DNA (1 μg/ml). (**E** and **F**) Immunoblotting analysis of the indicated proteins in L929 and U937 cells stimulated with poly(I:C) (1 μg/ml). (**G** and **H**) IRF3 phosphorylation and dimerization were analyzed by immunoblotting In L929 cells stimulated with HT-DNA or poly(I:C). The numbers above the p-IRF3 blots represent the grayscale intensity ratio of p-IRF3/IRF3 in **C**–**H**. (**I–L**) Immunofluorescence staining of IRF3 and p-IRF3 in Hs27 cells under HT-DNA or poly(I:C) stimulation. (**I** and **J**) Images were acquired with a Zeiss 880 confocal microscope. Scale bar, 10 μm. (**K** and **L**) Quantitative analysis of IRF3 nuclear translocation. *n* = 270 cells for each group. (**M** and **N**) Immunoblotting analysis of fractionated lysates from the cells under HT-DNA or poly(I:C) stimulation. α-Tubulin and histone H3 blots were used as controls for cytoplasmic and nuclear fractions, respectively.

Next, we investigated how indomethacin suppresses nucleic acid-stimulated IFN production. We found that indomethacin did not inhibit cGAMP synthesis in response to HT-DNA stimulation (Figure 5A) but significantly suppressed cGAMP-stimulated *Ifnb* expression (Figure 5B), suggesting that the inhibitory effect of indomethacin may be achieved by suppressing the downstream events of cGAMP. In response to cGAMP treatment, the endoplasmic reticulum protein STING (also known as MITA, MPYS, or ERIS) (Ishikawa and Barber, 2008; Jin et al., 2008; Zhong et al., 2008; Sun et al., 2009) is activated and promotes the downstream effectors TANK-binding kinase 1 (TBK1) and IRF3 to produce type I IFN (Bowie, 2012). However, IRF3 and TBK1 phosphorylation levels were not significantly affected by indomethacin upon HT-DNA stimulation in L929 and U937 cells (Figure 5C and D). Similarly, indomethacin had a marginal effect on the phosphorylation of IRF3 under poly(I:C) stimulation in L929 and U937 cells (Figure 5E and F). Moreover, IRF3 dimerization in response to HT-DNA or poly(I:C) was not affected by indomethacin in L929 cells (Figure 5G and H). Interestingly, the nuclear translocation of phosphorylated IRF3, a critical step for its function as a transcription factor, was significantly hindered by indomethacin (Figure 5I–L). The isolation of the cytosolic and nuclear fractions, followed by western blotting analysis, confirmed that indomethacin treatment prevented the translocation of IRF3 into nucleus (Figure 5M and N). Collectively, our data suggest that indomethacin inhibits both cytosolic DNA- and RNA-induced type I IFN production mainly through preventing the nuclear translocation of phosphorylated IRF3.

### Indomethacin suppresses self-DNA-stimulated autoimmunity in an AGS mouse model

**Figure 6:**
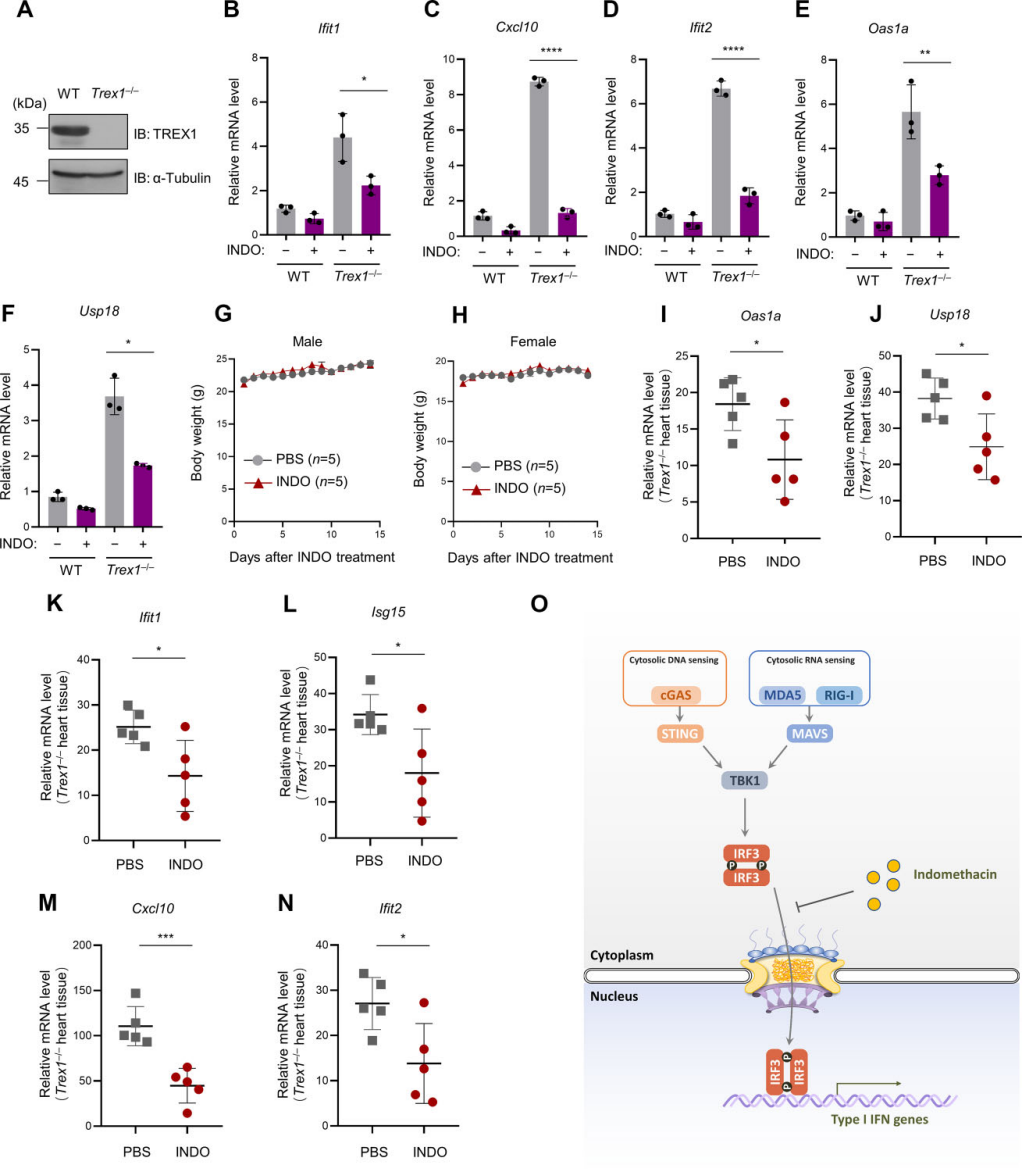
Indomethacin suppresses self-DNA-stimulated autoimmunity in an AGS mouse model. (**A**) Wild-type and *Trex1*^–/–^ bone marrow cells were validated by western blotting. (**B**–**F**) Relative mRNA levels of the indicated ISGs were analyzed by qPCR in wild-type and *Trex1*^−/−^ bone marrow cells treated with or without indomethacin (200 μM). *n* = 3 biological replicates. (**G** and **H**) Wild-type C57BL/6 mice were treated with indomethacin (3 mg/kg) twice a day for 2 weeks. Body weight was measured every day. (**I**–**N**) *Trex1*^–/–^ mice were given indomethacin (i.p., 1 mg/kg; *n* = 5) or PBS (*n* = 5) twice a day for 7 days. Relative mRNA levels of the indicated ISGs in mouse hearts were analyzed by qPCR. (**O**) A schematic describing the mechanisms by which indomethacin inhibits nucleic acid-stimulated type I IFN production. Data are presented as mean ± SD. *P < 0.05, **P < 0.01, ***P < 0.001, ****P < 0.0001, Welch's *t*-test (**F**) or Student's *t*-test (**B**–**E** and **I**–**N**).

We further investigated the potential of indomethacin as a treatment for nucleic acid-induced autoimmune responses. Bone marrow cells were isolated from both wild-type and *Trex1*^–/–^ mice (Figure 6A). Compared with wild-type bone marrow cells, *Trex1*^–/–^ bone marrow cells expressed higher mRNA levels of ISGs, which were significantly inhibited upon treatment with indomethacin (Figure 6B–F). To assess the potential toxicity of indomethacin, we treated mice with indomethacin (3 mg/kg) via intraperitoneal (i.p.) injection twice a day for 2 weeks, which did not lead to significant reduction in body weight (Figure 6G and H). We then evaluated the *in vivo* therapeutic effect of administrating indomethacin (1 mg/kg, i.p.) twice a day for 7 days. Our data showed that indomethacin significantly inhibited the mRNA levels of ISGs in the heart tissues of *Trex1*^–/–^ mice (Figure 6I–N). Thus, indomethacin treatment strongly inhibits ISG expression in bone marrow cells and heart tissues, suggesting that indomethacin is effective in controlling cytoplasmic DNA-mediated autoimmune responses. Overall, our study reveals a novel function of indomethacin in controlling type I IFN production by regulating IRF3 nuclear translocation and provides a potential treatment for cytosolic nucleic acid-mediated autoimmune diseases (Figure 6O).

## Discussion

Type I IFNs are the major cytokines involved in the host defense against viruses and other pathogens (Stetson and Medzhitov, 2006). Despite their critical roles in virus elimination, aberrant or excessive type I IFN production results in autoimmune conditions, termed interferonopathies (Crow, 2011; Lee-Kirsch, 2017; Crow and Stetson, 2022). Therefore, targeting the production of IFNs provides a potential treatment for these IFN-driven diseases.

In the current study, we screened several NSAIDs that are used as first-line clinical medications to identify their potentials in restraining type I IFN production. We observed that indomethacin, a NSAID belonging to the acetic acid derivatives, suppressed both cytoplasmic DNA- and RNA-induced IFN expression. Furthermore, we demonstrated that indomethacin achieved the inhibition of IFN expression by preventing the nuclear translocation of IRF3. Previously, we reported that another NSAID aspirin blocks cGAS activity by acetylating residues Lys384, Lys394, and Lys414 of cGAS (Dai et al., 2019), with a distinct mechanism of action in the inhibition of the cGAS–STING pathway. Aspirin directly suppresses cGAS and thus exhibits promising potential in treating cGAS-driven autoimmune diseases, such as AGS. This study provides additional strategies for treating autoimmune conditions with different pathogenesis. For example, SAVI is caused by STING mutations and thus inhibitors targeting STING or its downstream events are critical. Through inhibiting IRF3 function, indomethacin serves as a potential therapeutic drug for SAVI, which may not benefit from cGAS inhibitors.

As a member of the IRF family, IRF3 plays a critical role in promoting the expression of type I IFNs (Sato et al., 2000; Yanai et al., 2018). The activation of IRF3 is initiated by various signals, including viral infection, bacterial infection, and cellular stress (Taniguchi et al., 2001; Tamura et al., 2008). It is known that IRF3 activation includes at least three sequential steps: phosphorylation, dimerization, and nuclear translocation (Lin et al., 1998, 1999). Previous studies focused on the regulation of phosphorylation and dimerization of IRF3 (Petro, 2020), and the mechanism and the regulation of IRF3 nuclear translocation remain unclear. Some researchers suggest that nuclear transport receptors, such as Karyopherin α and β family proteins, facilitate the entry of IRF3 into the nucleus (Cai et al., 2020; Shen et al., 2021). It is possible that indomethacin modulates the cellular nucleocytoplasmic transport, thereby suppressing the production of type I IFNs. To validate this hypothesis, further studies are necessary. Because both intracellular DNA- and RNA-triggered signaling cascades converge on the activation of IRF3, the blockade of IRF3 could be a highly efficient means to regulate the inappropriate activation of both signaling pathways. Our study reveals the previously unknown function of indomethacin, i.e. it can reduce the expression of IFNs through targeting IRF3 nuclear translocation.

The effective treatment for AGS is still lacking. More importantly, it was reported that 81% of AGS patients with *TREX1, RNASEH2A*, and *RNASEH2C* mutations die by the age of 10 (Rice et al., 2007). Thus, it is crucial to develop safe therapeutical methods for treating children with AGS. Over 60 years ago, indomethacin was identified as an anti-inflammatory and analgesic medication with potent effects (Ballabio et al., 1963; Hart and Boardman, 1963; Smyth et al., 1963; Winter et al., 1963). Several studies have demonstrated the safety and effectiveness of indomethacin in treating children's diseases, such as patent ductus arteriosus and juvenile idiopathic arthritis (Hashkes and Laxer, 2005; Khuwuthyakorn et al., 2018; Ziesenitz et al., 2022). Our finding suggests indomethacin as a potential therapeutic drug, or at least a model compound for designing further pharmaceutical strategies, to treat AGS in children and possibly other type I interferonopathies.

## Material and methods

### Animals

*Trex1* ^+/–^ mice (C57BL/6) were obtained from Cancer Research UK (Morita et al., 2004; Yang et al., 2007). All animal experiments were conducted in compliance with the National Institutes of Health (NIH) Guide for the Care and Use of Laboratory Animals and were approved by the Institutional Animal Care and Use Committee. *Trex1*^–/–^ mice (3 weeks old) were injected (i.p.) with indomethacin (1 mg/kg) twice a day for 7 days. The total RNA was extracted from mouse hearts and the ISG mRNA levels were analyzed by qPCR.

### Cell lines

L929 is a murine cell line derived from the subcutaneous connective tissue. U937 is a human monocytic cell line. Hs27 is a human fibroblast cell line derived from the foreskin tissue.

### Cell culture and transfection

U937 cells were differentiated using phorbol myristate acetate (0.1 μM) for 36 h before any experiments. Human primary macrophages were differentiated from PBMCs using recombinant human granulocyte–macrophage colony-stimulating factor (GM-CSF) (25 ng/ml) for 7 days. We obtained peripheral blood from healthy donors with the help of Jiaxing Central Blood Station, where healthy blood donors were recruited according to the National Guide for Blood Donation (GB18467-2011). The isolation of PBMCs was performed under the approval of the Institutional Ethics Committee (AF/SC-08/02.40). Bone marrow-derived macrophages (BMDMs) were differentiated from bone marrow cells using recombinant mouse M-CSF (25 ng/ml) for 7 days. U937 cells, L929 cells, and human primary macrophages were cultured in RPMI-1640 medium (10% fetal bovine serum, 2 mM L-glutamine, and 1% penicillin–streptomycin). A549 cells and BMDMs were cultured in Dulbecco's modified Eagle medium (10% fetal bovine serum, 2 mM L-glutamine, and 1% penicillin–streptomycin). All the cell lines were tested and confirmed to be free of mycoplasma.

HT-DNA, poly(I:C), and ISD were transfected with Lipofectamine 2000 according to the manufacturer's instructions. For cGAMP stimulation, the cells were incubated with cGAMP at 37°C for 30 min in permeabilization buffer (50 mM HEPES, pH 7.0, 3 mM MgCl_2_, 100 mM KCl, 85 mM sucrose, 0.2% bovine serum albumin, 0.1 mM dithiothreitol, 1 mM adenosine triphosphate, 0.1 mM guanosine triphosphate, and 1 μg/ml digitonin).

### Antibodies and chemical reagents

The antibodies and chemical reagents used in this study are listed in Supplementary Table S1.

### RNA interference and qPCR

Cells were transfected with siRNA at a concentration of 100 nM for 48 h. Mouse si*Trex1*, human si*ADAR1*, and control siRNA were synthesized by GenePharma and RiboBio. Sequences are listed in Supplementary Table S2. The transfection of siRNA was performed with Lipofectamine RNAiMAX.

RNA was extracted using TRI reagent (Sigma). The extracted RNA was then reverse-transcribed using the Prime-Script RT Reagent Kit (TaKaRa). qPCR was conducted using SYBR Green Mix (A25742, Applied Biosystems), and primer sequences are provided in Supplementary Table S3.

### Cell viability assay

Cells were treated with indomethacin at the specified concentration for 24 h. After that, cell viability was measured by Cell Proliferation Assay kit (G3580, Promega), following the manufacturer's instructions.

### cGAMP quantitative analysis

After HT-DNA stimulation, cGAMP was isolated using an extraction solvent consisting of 40% methanol, 40% acetonitrile, and 20% water (*v*:*v*:*v*). The quantification of cGAMP was conducted using a triple-quadrupole mass spectrometer (Waters Xevo TQ-S), as previously described (Zhao et al., 2022).

### ELISA

The production of IFN-β in the cell culture medium was measured using IFN-β ELISA kits in accordance with the manufacturer's protocol (NEOBIOSCIENCE; cat# EMC016 for murine IFN-β and cat# EHC026 for human IFN-β).

### Immunoblotting

Cells were washed twice with cold phosphate-buffered saline (PBS) and lysed using a lysis buffer consisting of 20 mM Tris–HCl (pH 7.5), 0.5% NP-40 (AR0107, Boster Biological Technology), 250 mM NaCl, 3 mM EGTA, and 3 mM EDTA. Before use, a protease inhibitor cocktail (HY-K0010, MCE) was added to the lysis buffer. The cell lysates were then suspended for 20 min at 4°C, followed by centrifugation at 20000× *g* for 10 min at 4°C. The supernatants were collected and mixed with 5× sodium dodecyl sulfate (SDS) loading buffer and boiled. The samples were separated using SDS–polyacrylamide gel electrophoresis and transferred to polyvinylidene fluoride membranes. The membranes were blocked and incubated with the indicated antibodies. Finally, the proteins were visualized using ECL (ThermoFisher Scientific). Nuclear and cytosolic proteins were isolated using the Nuclear–Cytosol Extraction Kit (P1200, APPLYGEN).

### IRF3 dimerization assay

Cells were washed twice with cold PBS and lysed with lysis buffer containing 20 mM Tris–HCl (pH 7.5), 150 mM NaCl, 10% glycerol, 0.5% NP-40, and 1 mM Na_3_VO_4_. Protease inhibitor cocktail was added to the lysis buffer before use. The cell lysates were incubated on ice for 15 min and vortexed for 10 sec every 5 min. Centrifugation was performed at 1000× *g* for 5 min at 4°C. The supernatants were collected and centrifuged at 12000× *g* for 10 min at 4°C. A portion of the supernatants were mixed with 0.4% DOC-Na_2_ and 5× native loading buffer (300 mM Tris–HCl, pH 6.8, 0.1% bromophenol blue, and 50% glycerol) for native gel electrophoresis.

### Immunofluorescence

Hs27 cells were grown on coverslips in 24-well plates and treated with indomethacin for 24 h. After treatment, the cells were transfected with HT-DNA or poly(I:C) for 6 h. The cells were then fixed with 4% paraformaldehyde for 20 min at room temperature and permeabilized with 0.3% Triton X-100 for 10 min on ice. Blocking was performed using 5% normal goat serum for 1 h. The cells were incubated with anti-IRF3 and anti-phospho-IRF3 antibodies overnight at 4°C. Alexa Fluor 488-conjugated (A11029) and 647-conjugated (A21245) secondary antibodies (ThermoFisher Scientific) were incubated for 1 h before counterstaining with Hoechst. Images were acquired using a ZEISS LSM 880 (Zeiss) confocal microscope.

### Statistical analyses

Data are presented as mean ± SD. The statistical analyses were performed, and graphs were generated by GraphPad Prism.

## Supplementary Material

mjae015_Supplemental_File

## Notes

**Author contributions:** T.L. and T.X. supervised the project; T.L., T.X., and M.W. designed the experiments; M.W., T.X., X.-W.L., S.-C.Y., L.G., W.X., and H.C. performed studies in mice and experiments in cells; D.L., J.P., and M.Z. performed mass spectrometry for cGAMP; M.W., Z.-L.G., L.-M.S., and L.C. acquired images using a ZEISS LSM 880 confocal microscope; T.Z., X.-Q.Z., S.-Z.J., J.L., and Q.-Y.H. analyzed and validated the data; T.L., T.X., and M.W. wrote the manuscript.
