# Supplementary material for: Indomethacin restrains cytoplasmic nucleic acid-stimulated immune responses by inhibiting the nuclear translocation of IRF3
Source: J Mol Cell Biol. 2024 Apr 5;16(4):mjae015. doi: 10.1093/jmcb/mjae015 (PMC11472148; doi:10.1093/jmcb/mjae015)
Supplement: mjae015_Supplemental_File [file mjae015_supplemental_file.pdf]

Supplementary Table S1 Antibodies and Chemical Reagent

ANTIBODIES	SOURCE	IDENTIFIER
anti-TREX1	BD Transduction Laboratories	611986
anti-ADAR1	Abcam	ab88574
anti-IRF3	Abcam	ab68481
anti-human MDA5	Abcam	ab79055
and anti-human phospho-IRF3	Abcam	ab76493
anti-phospho-TBK1	Cell Signaling Technology	5483
anti-TBK1	Cell Signaling Technology	3504
anti-mouse phospho-IRF3	Cell Signaling Technology	4947
anti-mouse MDA5	Cell Signaling Technology	5321
anti- MX1	Cell Signaling Technology	37849s
anti-USP18	Cell Signaling Technology	4813T
anti-mouse IRF3 dimer	Cell Signaling Technology	4302
anti-mouse MAVS	Santa Cruz Biotechnology	sc-365333
anti-human MAVS	Santa Cruz Biotechnology	sc-166583
anti-α-tubulin	Sigma-Aldrich	T5168
anti-ISG15	Proteintech	15981-1-AP
anti-GAPDH	Proteintech	10494-1-AP
anti-IRF3	Santa Cruz Biotechnology	sc-33641
anti-phospho-IRF3	Beyotime Biotechnology	AF1594

REAGENTS	SOURCE	IDENTIFIER
HT-DNA	Sigma-Aldrich	D6898
PMA	Sigma-Aldrich	524400
digitonin	Sigma-Aldrich	D141
poly(I:C)	Invivogen	tlrl-pic
cGAMP	Invivogen	tlrl-cga23
ATP	ThermoFisher Scientific	R0441
GTP	ThermoFisher Scientific	R0461
Recombinant human GM-CSF	R&D Systems	215-GM
recombinant mouse M-CSF	R&D Systems	416-ML
Naproxen	TOPSCIENCE	T1582
Oxaprozin	TOPSCIENCE	T0708
Flurbiprofen	TOPSCIENCE	T0291
Sulindac	TOPSCIENCE	T0459
Etodolac	TOPSCIENCE	T1002
Nabumetone	TOPSCIENCE	T1258
Mefenamic acid	TOPSCIENCE	T0890
Tolfenamic acid	TOPSCIENCE	T0784
Meclofenamate Sodium	TOPSCIENCE	T0260
Ketoprofen	Selleck	S1645
Lornoxicam	Selleck	S2047
Piroxicam	Selleck	S1713
Tenoxicam	Selleck	S2512
Indomethacin	Selleck	S1723
Etoricoxib	Selleck	S4651
Celecoxib	Selleck	S1261
Parecoxib	Selleck	S4656
Flufenamic acid	Selleck	S4268
Ibuprofen	Selleck	S1638
Acetaminophen	Selleck	S1634
Salicylamide	Selleck	S6404
Salicylic acid	Selleck	S4539
Diclofenac Sodium	Aladdin	D129332
Aspirin	OUHE technology	50-78-2

Supplementary Table S2 siRNA sequence

Gene	5’-3’
Mouse *Trex1*	ACCGACAGACUCACAUACUGCUGAA
Human *ADAR1*	GCAGAGTCAGCATATATGA

Supplementary Table S3 Primers of ISGs in qPCR assay

Gene	Forward primers (5’-3’)	Reverse primers (5’-3’)
Mouse *Cxcl10*	GCCGTCATTTTCTGCCTCA	CGTCCTTGCGAGAGGGATC
Mouse *Ifit1*	GAACCCATTGGGGATGCACAACCT	CTTGTCCAGGTAGATCTGGGCTTCT
Mouse *Isg15*	TGACTGTGAGAGCAAGCAGC	CCCCAGCATCTTCACCTTTA
Mouse *Hprt*	CAGTCCCAGCGTCGTGATTAG	AAACACTTTTTCCAAATCCTCGG
Mouse *Usp18*	TTGGGCTCCTGAGGAAACC	CGATGTTGTGTAAACCAACCAGA
Mouse *Ifit2*	ATGAGTTTCAGAACAGTGAGTTTAA	AACTGGCCCATGTGATAGTAGACCC
Mouse *Oas1a*	GCCTGATCCCAGAATCTATGC	GAGCAACTCTAGGGCGTACTG
Mouse *Ifnb*	TCCGAGCAGAGATCTTCAGGAA	TGCAACCACCACTCATTCTGAG
Human *IFI27*	TGCTCTCACCTCATCAGCAGT	CACAACTCCTCCAATCACAACT
Human *IFI44*	TTTTCGATGCGAAGATTCACTGG	CCTGATGCGTTACATGCCCTT
Human *ISG15*	CGCAGATCACCCAGAAGATCG	TTCGTCGCATTTGTCCACCA
Human *GAPDH*	GAGTCAACGGATTTGGTCGT	TTGATTTTGGAGGGATCTCG
Human *IFNB*	AGGACAGGATGAACTTTGAC	TGATAGACATTAGCCAGGAG
